# The CARESSES Randomised Controlled Trial: Exploring the Health-Related Impact of Culturally Competent Artificial Intelligence Embedded Into Socially Assistive Robots and Tested in Older Adult Care Homes

**DOI:** 10.1007/s12369-021-00781-x

**Published:** 2021-04-23

**Authors:** Chris Papadopoulos, Nina Castro, Abiha Nigath, Rosemary Davidson, Nicholas Faulkes, Roberto Menicatti, Ali Abdul Khaliq, Carmine Recchiuto, Linda Battistuzzi, Gurch Randhawa, Len Merton, Sanjeev Kanoria, Nak-Young Chong, Hiroko Kamide, David Hewson, Antonio Sgorbissa

**Affiliations:** 1 University of Bedfordshire, Park Square Campus, Luton, LU1 3JU UK; 2 Advinia Health Care Limited LTD, 314 Regents Park Road, London, N3 2JX UK; 3 Department of Informatics, Bioengineering, Robotics and Systems Engineering, University of Genova, Via all’Opera Pia 13, 16145 Genova, Italy; 4 School of Science and Technology, Orebro University, Orebro, Sweden; 5 Japan Advanced Institute of Science and Technology, 1-1 Asahidai, Nomi, Ishikawa 923-1292 Japan; 6 Nagoya University, Furocho, Chikusaku, Nagoya, Aichi 464-8601 Japan

**Keywords:** CARESSES, Mental health, Socially assistive robots, Experimental trial, Older adults, Cultural competence

## Abstract

This trial represents the final stage of the CARESSES project which aimed to develop and evaluate a culturally competent artificial intelligent system embedded into social robots to support older adult wellbeing. A parallel group, single-blind randomised controlled trial was conducted across older adult care homes in England and Japan. Participants randomly allocated to the Experimental Group or Control Group 1 received a Pepper robot for up 18 h across 2 weeks. Two versions of the CARESSES artificial intelligence were tested: a fully culturally competent system (Experimental Group) and a more limited version (Control Group 1). Control Group 2 (Care As Usual) participants did not receive a robot. Quantitative outcomes of interest reported in the current paper were health-related quality of life (SF-36), loneliness (ULS-8), and perceptions of robotic cultural competence (CCATool-Robotics). Thirty-three residents completed all procedures. The difference in SF-36 Emotional Wellbeing scores between Experimental Group and Care As Usual participants over time was significant (F[1] = 6.614, sig = .019, η_p_^2^ = .258), as was the comparison between Any Robot used and Care As Usual (F[1] = 5.128, sig = .031, η_p_^2^ = .146). There were no significant changes in SF-36 physical health subscales. ULS-8 loneliness scores slightly improved among Experimental and Control Group 1 participants compared to Care As Usual participants, but this was not significant. This study brings new evidence which cautiously supports the value of culturally competent socially assistive robots in improving the psychological wellbeing of older adults residing in care settings.

## Introduction

Health and social care sectors worldwide are facing rising pressures due to increased demand associated with aging populations, the many complex chronic conditions associated with this population, and now the impact of Covid-19. Therefore, exploring technological solutions that may alleviate these pressures in a safe, ethical, acceptable and effective manner is vital.

The evidence regarding the impact of assistive robotics for older adults with care needs is emerging and, in relation to psychological wellbeing, appears encouraging. Abdi, Al-Hindawi, Ng, and Vizcaychipi’s [1] scoping review of socially assistive robots (SARs) within older adult healthcare settings assessed a range of outcomes from 33 studies, totalling 1574 participants and 11 robots. Twenty-eight of these studies reported positive outcomes in relation to assisting older adults with cognitive training, companionship, social facilitation and physiological therapy. However, the authors conclude that while the evidence for SARs is promising, many studies had methodological issues and most only focused upon small robots, in particular the PARO seal robot.

Systematic reviews by Pu, Moyle, Jones, and Todorovic [2] and Abbott et al. [3] reached similar conclusions. The former examined the effectiveness of SARs for older adults from data extracted from nine randomized controlled trials, concluding that, overall, social robots do have the potential to reduce anxiety, agitation, loneliness and improve quality of life for older adults despite non-significant results in their meta-analysis. Abbott et al.’s [3] review of qualitative and quantitative evidence in relation to the use of robopets (small animal‐like companion robots) in improving older adults’ wellbeing similarly reported that, on the whole, there is evidence of positive impact in relation to reduced agitation and loneliness despite non-significance within their meta-analysis. Both reviews however also highlighted a large proportion of evidence has to date been produced by studies using the PARO robot only and that many studies were had a high risk of bias, particularly in relation to allocation concealment and blinding.

The current study, part of the CARESSES project, attempted to build on the current evidence-base by conducting an exploratory RCT using a novel culturally competent and autonomous artificial intelligence system [4, 5] developed earlier in the project, and embedded into the Pepper humanoid robot using (which has to date been largely overlooked by other similar trials to date) within older adult care settings in the UK and Japan. It is the first to employ a cross-national trial design, and among the first to explore the impact of culturally competent artificial intelligence embedded into social robotics for enhancing health and wellbeing. The importance of cultural competence in improving patient outcomes is widely reported, particularly within the nursing literature [6, 7]. The concept refers to the ability of services to effectively meet and leverage the cultural and communication needs of clients [6]. Yet despite both its importance for enhancing patient-centred care and considerable debate around the conceptualisation and construction of ‘cultural robotics’ [8–11], the concept has never previously been implemented and evaluated for its utility using a randomised controlled trial design within healthcare settings. Cultural competence has also been linked with improved patient satisfaction [12] and acceptance [13, 14], which are two further key aspects of successful assistive robotics solutions [15–17].

The CARESSES project was formed on the aforementioned points, namely that producing an artificial intelligence that embeds cultural competence to achieve patient-centredness should boost SARs’ overall quality of user interactions, boost user acceptance, and that further research into producing safe, acceptable and effective socially assistive robotic solutions is needed so to help reduce the strain on the older adult care sector. The current paper specifically presents the trial’s quantitative results in relation to the intervention’s impact upon health-related quality of life, mental health and loneliness, when culturally competent socially assistive robots are used to supplement existing older adult care in long-term residential care settings.

## Methods

### Study Design and Setting

The CARESSES project involved a mixed-method, single-blind (among participants receiving a robot only), parallel-group experimental trial was employed within long-term older adult care homes in England and Japan during 2019. A pre-trial pilot study was first employed during late 2018 in one UK-based care home (which did not feature in the main trial). This established that our planned procedures were likely to be feasible and acceptable, and led to several procedural and technical improvements that were subsequently employed to enhance the main trial’s procedures.

The University of Bedfordshire’s Research Ethics Committee approved the UK-based study (Ref: UREC130) whereas the Human Subject Research Ethics Review Technical Subcommittee of the Japan Advanced Institute of Science and Technology Life Science Committee approved the Japan-based study (Ref: 30–001). Full details on how ethical considerations, a key component of the overall project, were identified and managed can be found elsewhere [18–20]. An overview of the methodological approach follows below although full details pertaining to methodological procedures (including the full suite of data collection measures used) have been published elsewhere [20].

### Participants

Participants were recruited on the following basis: they were aged ≥ 65 years; resided in a single occupancy bedroom / bedroom area within their care home; were unlikely to express aggression towards themselves, the robot, and/or the researcher; possessed sufficient cognitive competence and sufficient physical health; and were able to verbally communicate in English (UK site only) or Japanese (Japan site only). Residents who self-identified themselves as primarily belonging to the English or Indian culture were recruited from UK-based care homes predominantly owned by Advinia Health Care (a full research partner in the project), whereas the Japanese participants were recruited from the HISUISUI assisted living facility in Japan.

To determine eligibility, care home staff first nominated residents (using initials only to protect anonymity) who they believed met the inclusion criteria. The research team then conducted brief structured interviews with staff who made nominations using the interRAI-Long Term Care Facility ‘Cognitive Performance Scale’ and ‘Aggressive Behaviour Scale’ sub-scales [21] to assess cognitive competence and aggressiveness, and the FRAIL-NH scale [22] to assess frailty (a proxy to physical health). Residents who passed screening were then approached by a familiar care home staff member and introduced to a researcher who proceeded to invite the resident to participate.

### Allocation and Blinding

Participants were allocated to one of following three groups using random sampling stratified by gender: 1. Experimental group (utilizing a CARESSES experimental robot); 2. Control Group 1 (utilizing a CARESSES control robot); and 3. Control Group 2 (Care As Usual only). Participants who received a robot were blinded to which type of robot they received (the experimental or control robot). Care home staff were also blinded and there were no circumstances under which unblinding was permissible.

### Trial Preparation

All research staff were subject to Disclosure & Barring Service checks (in the UK) and criminal record checks (in Japan). Researchers also completed a series of ethics and methods training, had weekly supervisory meetings and team support on an on-going basis. Care home staff were prepared via a brief presentation regarding what to expect during testing procedures, to request they continue their jobs as usual, to not rely upon the robot and—to help protect staff morale and concerns about the implications of technological interventions—reminded that the project is exploring whether and how social robots may support outcomes in conjunction with current care rather than replacing care. Leaflets that contained this information were also made available.

Technical preparation and set-up procedures lasting approximately 1 h took place within each participant’s bedroom prior to the first test either in the presence of the resident or without their presence if they preferred and provided consent for. The research team also had a brief preparatory meeting with participants allocated to receive a robot so to answer any remaining questions and to collect key personal information required to customise the robot (e.g. name, age and the contact information of close family members the robot could use to contact if the resident wished).

### Boosting Standardisation

Several procedures took place to boost procedural standardisation between the UK and Japan-based sites. This included regular joint planning meetings, a jointly produced, detailed protocol, using the same data collection instruments (including pre-existing validated Japanese translations where possible), researchers from both sites undergoing the same series of training procedures, and step-by-step manuals to aid implementation of procedures.

### Interventions

Participants allocated to the Experimental Group or Control Group 1 received a robot for two weeks. The robot hardware used was ‘Pepper’, a robot manufactured by SoftBank Robotics (a study partner). It is 4 ft tall, weighs 63 lb and, due to its artificial appearance, is not disadvantaged by the Uncanny Valley effect [23].

The CARESSES Experimental Robot represented our best effort of producing robotic cultural competence. This involved (a) the robot being made aware of the particular participants’ cultural background; (b) pre-loading and employing the appropriate Cultural Knowledge Base (CKB) during testing (for which three such databases were developed for the English, Indian and Japanese cultures); (c) initially tailoring its interactions to a culture-specific level and then, during the second half of testing only, shifting towards more personalised interactions after learning more about the participants’ individual preferences and values; and (d) propagating its learning of the participant in one particular area automatically to other related areas for improved predictions about the participants’ values and preferences.

The CARESSES Control Robot represented our attempt at ensuring clinical equipoise through producing a robot that was less culturally competent but also not culturally incompetent (where it might be reasonably likely to expect harm). This robot was therefore not pre-aware of the participant’s cultural background, it did not propagate its learning and it pre-loaded a generic and more limited CKB not tailored for any particular cultural profile. However, it did tailor its interactions to become more personalised over time.

An example to illustrate the differences between these versions is that for a participant who primarily identifies with the English culture, the Experimental Robot would load its English CKB upon first meeting the individual, initiate a greeting that is likely to be valued (e.g. hand waving), and to begin conversing in topics of conversation likely to be valued as encoded within the CKB (e.g. talking about football, Hollywood movies, Western music, World War 2). Conversations could then lead to the robot asking whether the participant might like to watch videos, listen to music or play a memory game associated with such interests. However, for the Control Robot, upon initially meeting the participant, would instead take a guess at an appropriate initial greeting gesture (e.g. a bow), and also make less accurate guesses about what topics of conversation are likely to be valued (e.g. asking the English participant whether he/she enjoys Sumo Wrestling or Bollywood movies), also enquiring whether the participant might wish to watch, listen or play a game associated with these topics. During the second week of testing, both versions adjust to the specific responses obtained in relation to conversation topics (e.g. if the participant states he/she does not like football, then both versions remember this and explore other sports which he/she may instead value). If the participant agrees that he/she likes Sumo Wrestling, the Experimental robot propagates this learning to also guess that he/she might also value other aspects of Japanese culture, whereas the Control Robot does not form such connections. A detailed explanation as to how the conversational interactions were developed and implemented during the trial stage has been previously described [24, 25]

The development of the CARESSES experimental and control robots required finding innovative technological solutions in order to embed the robots with the required Artificial Intelligence, enabling the robot to interact with the participants using its sensors and algorithms for data processing and reasoning. Indeed, the basic version of Pepper provides basic functionalities for interacting with people, but they need to be orchestrated through specific cognitive processes in order to enable a natural and engaging long-term interaction (given the aim was not to implement Wizard-of-Oz experiments but rather fully autonomous behaviour). A full explanation on how the artificial intelligence for the experimental and control robots was developed for the purpose of testing and evaluation has been previously described [26].

### Testing Procedures

Participants allocated to receive a robot had six sessions with the robot spread across two weeks at convenient times for the residents. Each session lasted for up to 3 h, with participants free to use the robot as much or as little as they wished during these times. The first session involved training the participant on how to use the robot and access the various functionalities, how to communicate with it, how to request assistance, and to answer remaining questions. The subsequent sessions involved the participants using the robot independently and privately (although for safety reasons, sessions were audio–video monitored by researchers nearby which participants were aware of). This involved the robot initially being placed near to the resident (within 3 feet) who could then specify further how close they wished the robot to remain positioned. Participants then used the robot to engage in conversation, listen to music, watch videos, play games, and to contact loved ones via text, video messages, video or audio calls. For safety reasons, during each session, the robot would remain in its initial position and not move around to follow the resident, although it would swivel to face the resident as necessary. After testing was complete, the research team spent additional time to support participants in case they expressed any sadness or distress from missing the robot. Control Group 2 (Care As Usual) participants did not engage in any tests and instead continued to receive their usual level and type of care.

### Data Collection

The quantitative measures of health and wellbeing applied during 1–1 structured interviews with residents at baseline and post-intervention were: (1) Short Form (36) Health Survey version (SF-36) [27]. This is a widely used and validated health survey measuring eight dimensions of mental and physical health. This was completed by all participants; (2) Short Form UCLA Loneliness Scale (ULS-8) [28]. This is a widely used brief and validated measure of loneliness and was also completed by all participants.

The Cultural Competence Assessment Tool–Robotics (CCATool-Robotics) tool was employed to assess perceptions of robotic cultural competence. This is an adapted version of the RCTSH Cultural Competence Assessment Tool (CCATool) [29]. This was completed by participants who received a robot at the one-week stage and post-intervention. Measurements were taken during these time periods to assess to what degree perceptions of cultural competence changed after personalisation had occurred during week 2. All data were collected between February and November 2019.

### Data Analysis

Data were first checked for accuracy with any discrepancies audited, discussed and resolved. There were two instances of missing SF-36 data. This scale tolerates small amounts of missing data due to its use of mean average subscales [27] and as such no adjustment or imputation was needed. There was one instance of missing data regarding the ULS-8 data for which case mean substitution was used as it was contextually appropriate to do so [30]. There were no missing data for the CCATool data.

Cronbach’s Alpha scores were good. Specifically, SF-36 scored 0.758 and 0.741, while ULS-8 scored 0.756 and 0.830 at baseline and post-intervention respectively.

Kolmogorov–Smirnov Normality Tests were conducted so to establish the normality of distribution among the dependent variables. Further, observation of scale variable differences between means and medians, kurtosis, skew values and histogram distributions were also observed. Overall, the data was assessed to be non-normally distributed and therefore non-parametric tests were applied. For within-group repeated analyses, Wilcoxon signed-rank tests were applied. Analysis of covariance (ANCOVA) tests were also carried out to measure and compare post-intervention outcome scores across a grouping variable (controlling for baseline scores). Grouping variables were constructed according to the group to which they were allocated to (Experimental Group, Control Group 1, Control Group 2). To expand the available analytical comparisons, an ‘Any Robot’ grouping variable was constructed which combined the data collected from the Experimental Group and Control Group 1 groups. Assessing the impact of perceived cultural competence on outcome change was made through assessing the differences in outcome scores between all available analytical comparisons (Exp Group vs Control Group 1, Exp Group vs Control Group 2, Control Group 1 vs Control Group 2, Exp Group + Control Group 1 [‘Any Robot’] vs Control Group 2).

Significance was set at *p* < 0.05, two-tailed. IBM SPSS Statistics v26 was used to run the analyses.

## Results

### Sample Characteristics

Nine care homes across England (n = 8) and Japan (n = 1) agreed to participate. Across these homes, 134 residents were nominated by care staff, 111 of whom passed screening. During initial recruitment procedures, it was observed that twelve further residents were unlikely to meet eligibility criteria (mainly due to perceived memory problems). Of the remaining 99 participants, 45 consented although ten subsequently dropped out prior to tests commencing. The remaining 35 participants commenced procedures although two participants (both allocated to control group 1) withdrew after 1 session (n = 1) and 2 sessions (n = 1). Thus, overall, 33 residents fully participated who identified themselves as primarily belonging to English (n = 15), Indian (n = 15) or Japanese (n = 3) cultures. The recruitment rate (eligible and invited / participated) was therefore 35.4%. The main reasons for declining participation were: ‘too tired’ (n = 14), no interest in project (n = 5), against the idea of robots in care homes (n = 3), too busy (n = 3) and family refused to consent (n = 3).

As can be seen in Tables 1 and 2, participants’ age ranged from 65—98 years (Mean = 81.9; SD = 9.82), the oldest of whom were Japanese (M = 86; SD = 9.54) followed by English (M = 83.67; SD = 10.3) and Indian participants (M = 79.4; SD = 9.36). The sample was predominantly female (n = 22), educational level was fairly equally distributed, and most participants were married at some point in their lives, with a large portion now widowed (n = 23). Participants were of multiple specific religious faiths which for analytical purposes were categorised as Christian (n = 13), Hindu (n = 14) or other (this mostly consisted of atheist or ‘non-religious’ people) (n = 6). A full overview of participant characteristics per group allocation can be found in Table 3.

**Table 1:** Sociodemographic frequencies—Overall and cultural grouping

	Age		Sex		Educational level			Marital status		Religiousness		
	Mean (SD)	Median	F N (%)	M N (%)	Primary or less N (%)	Secondary/High school N (%)	Professional/College N (%)	Widow N (%)	Not Widow N (%)	Not Religious N (%)	Slightly/ Moderately Religious N (%)	Very/Extremely Religious N (%)
Overall N = 33	81.94 (9.81)	81	22 (66.7)	11 (33.3)	11 (33.3)	12 (36.4)	10 (30.3)	23 (69.7)	10 (30.3)	7 (21.2)	16 (48.5)	10 (30.3)
English N = 15	83.67 (10.30)	83	9 (27.3)	6 (18.2)	7 (21.2)	5 (15.2)	3 (9.1)	11 (33.3)	4 (12.1)	4 (12.1)	10 (30.3)	1 (3.0)
Indian N = 15	79.40 (9.36)	80	10 (30.3)	5 (15.2)	3 (9.1)	5 (15.2)	7 (21.2)	10 (30.3)	5 (15.2)	0 (0.0)	6 (18.2)	9 (27.3)
Japanese N = 3	86.00 (9.54)	87	3 (9.1)	0 (0)	1 (3.0)	2 (6.1)	0 (0)	2 (6.1)	1 (3.0)	3 (9.1)	0 (0.0)	0 (0.0)

**Table 2:** Sociodemographic frequencies—Allocation and robot grouping

	Age		Sex		Educational level			Marital status		Religiousness		
	Mean (SD)	Median	F N (%)	M N (%)	Primary or less N (%)	Secondary/High school N (%)	Professional/College N (%)	Widow N (%)	Not Widow N (%)	Not Religious N (%)	Slightly/ Moderately Religious N (%)	Very/ Extremely Religious N (%)
All robots N = 23	80.65 (9.48)	81	14 (60.9)	9 (39.1)	5 (15.2)	9 (27.3)	9 (27.3)	16 (48.5)	7 (21.2)	5 (15.2)	13 (39.4)	5 (15.2)
Exp robot N = 12	79.50 (9.03)	79	7 (21.2)	5 (15.2)	1 (3.0)	5 (15.2)	6 (18.2)	8 (24.2)	4 (12.2)	3 (9.1)	8 (24.2)	1 (3.0)
Control robot N = 11	81.91 (10.23)	83	7 (21.2)	4 (12.1)	4 (12.1)	4 (12.1)	3 (9.1)	8 (24.2)	3 (9.1)	2 (6.1)	5 (15.2)	4 (12.1)
No robot N = 10	84.90 (10.44)	84	8 (24.2)	2 (6.1)	6 (18.2)	3 (9.1)	1 (3.0)	7 (21.2)	3 (9.1)	2 (6.1)	3 (9.1)	5 (15.2)

**Table 3:** Mean SF-36 subscale scores per time point by robot grouping

	SF 36 Subscales—Physical								SF 36 Subscales—Mental							
	Physical function		Role limitations—Physical		Pain		General health		Role limitations—Emotional		Energy and Fatigue		Emotional wellbeing		Social functioning	
	T0 Mean (SD)	T2 Mean (SD)	T0 Mean (SD)	T2 Mean (SD)	T0 Mean (SD)	T2 Mean (SD)	T0 Mean (SD)	T2 Mean (SD)	T0 Mean (SD)	T2 Mean(SD)	T1 Mean (SD)	T2 Mean (SD)	T0 Mean (SD)	T2 Mean (SD)	T0 Mean (SD)	T2 Mean (SD)
Any robot N = 23	34.32 (27.06)	32.83 (27.87)	63.32 (41.13)	60.60 (42.45)	69.57 (23.94)	65.43 (28.17)	65.22 (19.57)	59.13 (20.43)	86.96 (31.36)	76.09 (39.19)	56.30 (24.13)	58.48 (22.69)	78.78 (17.16)	82.43 (16.66)	80.43 (23.18)	76.63 (21.75)
Exp robot N = 12	32.45 (28.46)	26.25 (23.66)	58.85 (43.00)	61.98 (39.03)	67.08 (25.56)	68.33 (31.39)	70.42 (13.22)	59.17 (19.87)	86.11 (33.21)	90.28 (28.83)	57.92 (23.82)	60.00 (24.31)	83.00 (11.83)	89.33 (7.69)	83.33 (23.44)	73.96 (21.62)
Control robot N = 11	36.36 (26.65)	40.00 (31.38)	68.18 (40.45)	59.09 (47.79)	72.27 (22.95)	62.27 (25.31)	59.55 (24.23)	59.09 (22.00)	87.88* (30.81)	60.60* (44.27)	54.55 (28.82)	56.82 (21.83)	74.18 (21.19)	74.91 (20.64)	77.27 (23.60)	79.55 (22.55)
No robot N = 10	46.00 (30.62)	47.50 (37.21)	57.50 (47.21)	60.00 (44.41)	71.25 (35.46)	72.75 (32.13)	72.00 (22.63)	70.00 (24.94)	73.33 (37.84)	53.33 (47.66)	62.50 (18.60)	56.00 (22.46)	82.80 (12.66)	67.60 (29.96)	86.25 (17.13)	76.25 (25.99)

^*^p < .05 (Wilcoxon Signed Ranks Test); SF36 scores range from 0 to 100 with 100 representing perfect health

### Quantitative outcome measures

Table 4 describes the overall mean total scores of each outcome measure at initial assessment (either Baseline [T0] or after one week [T1] depending on tool) and post-intervention [T2] per group. SF-36 mental health total mean scores significantly decreased for the Care As Usual group (T0: M = 76.22 [SD = 16.51]; T2: M = 63.30 [SD = 25.3]; *p* < 0.05), whereas participants receiving a robot did not observe such a reduction, and, in the case of the Experimental group, a slight increase (T0: M = 77.59 [SD = 16.4]; T2: M = 78.39 [SD = 12.15]) was observed. For the SF-36 mental health subscales, small score improvements for the Experimental Group can also be seen for the ‘role limitations—emotional’ (i.e. the degree to which emotional health impacts upon one’s ability to conduct their usual roles) (T0: M = 86.11 [SD = 33.21]; T2: M = 90.28 [SD = 28.83]) and ‘energy and fatigue’ subscales (T0: M = 57.92 [SD = 23.82]; T2: M = 60.00 [SD = 24.31]). However, a significant decrease in the ‘role limitations—emotional’ scale was also observed for the Control group across the two time points (T0: M = 87.88 [SD = 30.81]; T2: M = 60.60 [SD = 44.27]; Z = − 2.041, sig = 0.041). A numerical although non-significant mean score increase was observed for the ‘emotional wellbeing’ subscale in the Experimental group (T0: M = 83.00 [SD = 11.83]; T2: M = 89.33 [SD = 7.69]) whereas a considerable numerical decrease was observed for the Care As Usual group (T0: M = 82.80 [SD = 12.66]; T2: M = 67.60 [SD = 29.96]). As seen in Tables 5 and 6, ANCOVA comparisons revealed that the relative improvement of ‘emotional wellbeing’ subscale scores between Experimental group participants and Care As Usual participants over time was significant (F[1] = 6.614, sig = 0.019, η_p_^2^ = 0.258), as was the comparison between Any Robot (i.e. participants in the Experimental or Control group) and Care As Usual (F[1] = 5.128, sig = 0.031, η_p_^2^ = 0.146). There were no significant changes in mean scores over time in SF-36 physical health subscales or with the ULS-8 loneliness scores. However, for the latter, the ‘Any Robot’, Experimental and Control groups experienced a slight reduction in loneliness severity between T0 and T2 (Exp group: T0: M = 14.9 [SD = 4.98]; T2: M = 14.3 [SD = 3.53], Control Group: T0: M = 18.8 [SD = 4.73]; T2: M = 17.2 [SD = 6.46]) whereas the Care As Usual group saw a slight increase in loneliness scores (T0: M = 15.7 [SD = 4.73]; T2: M = 16.5 [SD = 5.4]).

**Table 4:** Mean outcome scores per time point by robot grouping

	SF36 totals				ULS-8 totals^+^		CCATool totals	
	Physical health totals		Mental health totals					
	T0 Mean (SD)	T2 Mean (SD)	T0 Mean (SD)	T2 Mean (SD)	T0 Mean (SD)	T2 Mean (SD)	T1 Mean (SD)	T2 Mean (SD)
Any robot N = 23	58.11 (20.79)	54.50 (18.28)	75.62 (16.81)	73.41 (18.99)	16.80 (5.13)	15.75 (5.28)	20.17 (4.60)	21.87 (3.44)
Exp robot N = 12	57.20 (20.36)	53.93 (15.21)	77.59 (16.40)	78.39 (12.15)	14.9 (4.98)	14.3 (3.53)	20.83 (4.84)	22.42 (2.91)
Control robot N = 11	59.09 (22.20)	55.12 (21.91)	73.47 (17.79)	67.97 (23.84)	18.8 (4.73)	17.2 (6.46)	19.45 (4.44)	21.27 (4.00)
No robot N = 10	61.69 (27.92)	62.56 (30.41)	76.22* (16.51)	63.30* (25.30)	15.70 (4.73)	16.50 (5.40)	–	–

*p < .05 (Wilcoxon Signed Ranks Test); SF36 scores range from 0 to 100 with 100 representing perfect health; ULS-8 scores range from 8 to 32 with 32 representing severe loneliness; CCATool scores range from 0 to 28 with 28 representing highest perceived cultural competence

**Table 5:** ANCOVA results for outcome scores for participants in 'Experimental robot' group vs 'Care as usual' group

Outcome variable	F	df	Sig	Partial eta squared
SF36 Physical function	1.352	1	.259	.066
SF36 Role limitations physical	.007	1	.934	.000
SF36 Role limitations emotional	3.993	1	.060	.174
SF36 Energy and fatigue	1.612	1	.220	.078
SF36 Emotional wellbeing	6.614	1	.019	.258
SF36 Social functioning	.031	1	.861	.002
SF36 Pain	.009	1	.924	.000
SF36 General health	2.095	1	.164	.099
SF36 Health change	.940	1	.345	.047
SF36 Physical total	.660	1	.427	.034
SF36 Mental total	3.836	1	.065	.168
ULS-8 Total scores^+^	1.250	1	.280	.072

^+^Japanese ULS-8 data excluded

**Table 6:** ANCOVA results for outcome scores for participants in 'Any Robot' group vs 'Care as usual' group

Outcome variable	F	df	Sig	Partial eta squared
SF36 Physical function	.512	1	.480	.017
SF36 Role limitations physical	.057	1	.813	.002
SF36 Role limitations emotional	1.203	1	.281	.039
SF36 Energy and fatigue	.848	1	.364	.027
SF36 Emotional wellbeing	5.128	1	.031	.146
SF36 Social functioning	.024	1	.878	.001
SF36 Pain	.665	1	.421	.022
SF36 General health	.928	1	.343	.030
SF36 Health change	.778	1	.385	.025
SF36 Physical total	.903	1	.349	.029
SF36 Mental total	2.251	1	.144	.070
ULS-8 Total scores^+^	1.163	1	.290	.041

^+^Japanese ULS-8 data excluded

Regarding perceptions of cultural competence, both versions of the robot produced fairly high levels of CCATool scores at both time points. However, the Experimental group’s robot performed better than the Control group’s robot at both time points in all but two of the subscales (Subscale B: Cultural Sensitivity at T2 and Subscale D: Cultural Competence at T1). CCATool scores increased between T1 and T2 for all scales within the Experimental group and all but in one scale for the Control group (Subscale D: Cultural Competence—T1: M = 4.91 [SD = 1.51, T2: M = 4.45 [SD = 1.51]). A full breakdown of CCATool scores can be seen in Table 7.

**Table 7:** CCATool total and subscales scores by robot grouping

	CCATool totals		CCATool subscale A		CCATool subscale B		CCATool subscale C		CCATool subscale D	
	T1 Mean (SD)	T2 Mean (SD)	T1 Mean (SD)	T2 Mean (SD)	T1 Mean (SD)	T2 Mean (SD)	T1 Mean (SD)	T2 Mean (SD)	T1 Mean (SD)	T2 Mean (SD)
Any robot N = 23	20.17 (4.60)	21.87 (3.44)	5.13 (1.49)	5.91 (1.16)	4.48 (1.73)	5.22 (1.51)	5.52 (1.47)	5.96 (0.98)	4.83 (1.44)	4.78 (1.20)
Exp robot N = 12	20.83 (4.84)	22.42 (2.91)	5.25 (1.54)	6.08 (1.08)	4.92 (1.73)	5.17 (1.19)	5.58 (1.88)	6.08 (0.90)	4.75 (1.42)	5.08 (0.79)
Control robot N = 11	19.45 (4.44)	21.27 (4.00)	5.00 (1.48)	5.73 (1.27)	4.00 (1.67)	5.27 (1.85)	5.45 (0.93)	5.82 (1.08)	4.91 (1.51)	4.45 (1.51)

CCATool total scores range from 0 to 28 with 28 representing highest perceived cultural competence; CCATool subscale scores range from 0 to 7 with 7 representing highest perceived cultural competence

## Discussion and Conclusions

While the findings are nuanced and are the product of an exploratory trial with several limitations, overall, the results cautiously provide further evidence in support of socially assistive robots being used to support older adults in care settings in a supplementary way, particularly in relation to mental health and emotional wellbeing.

Firstly, fairly large and positive score changes in the ‘emotional wellbeing’ subscale were observed in both the Any Robot group and the Experimental group, whereas no change in score was observed for the Control group and a fairly large decrease in scores was observed for the Care As Usual group. When assessing the magnitude of change in scores over time between groups, the differences between improved scores in the Any Robot group compared to the Care As Usual group, and between the Experimental group compared to the Care As Usual group, were significant. This implies that using a CARESSES robot (ideally the Experimental Robot) compared to not using any robot may indeed be likely to improve older adults’ emotional wellbeing, even only after a period of 2 weeks. Whether and to what degree this effect may hold or change over time is however currently unknown.

When examining the SF-36 mental health total scores, which may be argued to be a less precise way of measuring mental health than the ‘emotional wellbeing’ subscale alone [31, 32], the differences in score change over time between groups were close to meeting the statistical significance threshold (Experimental group vs Care As Usual group: F[1] = 4.249, sig = 0.054; Experimental group vs Control group: F[1] = 3.836, sig = 0.065). These findings lend further support to the notion that having access to a version of the CARESSES intervention over 2 weeks may be likely to protect older adults’ mental health compared to not using any robot, and that the Experimental robot is likely to be particularly effective at this.

The Experimental robot also produced better scores during testing on the ‘role limitation—emotional’ SF-36 subscale compared to the both the Control and Care As Usual groups. This scale measures how limited individuals are in conducting their usual activities because of emotional problems. As can be observed in Table 3, the Experimental group mean scores slightly increased, whereas the Control group’s scores significantly decreased (Z = − 2.041; sig = 0.041) and the Care As Usual group also observed a large decrease (although a non-significant one). These between-group differences were close to significance (Experimental group vs Care As Usual: F[1] = 3.993, sig = 0.06; Experimental group vs Control robot group: F[1] = 3.792, sig = 0.066). These findings therefore provide some further evidence that using a culturally competent socially assistive robot may be likely to protect against mental health problems, including those which impact upon one’s everyday activities.

An SF-36 measure that both the versions of the robot performed reasonably well in comparison to the Care As Usual group was ‘energy and fatigue’, with both robot groups showing a slight increase in scores over time whereas the Care As Usual group had a larger, although non-significant numerical decrease. The reasons for why the robots may have protected participants’ energy levels is difficult to interpret although it may be that gains in mental health and emotional wellbeing boosted energy (indeed, a significant correlation between emotional wellbeing and energy/fatigue can be observed: rho = 0.499; sig = 0.003).

The intervention was not able to produce any discernible impact in terms of physical health. These findings are unsurprising; the intervention was designed with a stronger focus on boosting mental health through culturally competent interactions rather than boosting physical health. The robots did offer participants the option to watch exercise videos and in a few cases the participants attempted to copy some of the basic exercises they were watching. However, clearly the offer of videos and providing tips about improving physical health was not effective in producing clear physical health improvements in such a short period of time and with what is a frail and physically disabled population. It should also be noted that there are limitations associated with the hardware given that the Pepper robot is not designed or able to provide any physical assistance. The Pepper hardware is also not able to dispense medication which in theory could have boosted medication adherence and thus physical health may have been improved. Furthermore, it may have been helpful for the system to have included reminders about taking medication; however, this feature was not made available during testing due to ethical concerns.

With regard to loneliness, ULS-8 baseline scores for the study samples were identified to be fairly high, which for this population is unsurprising [33, 34]. During testing, severity of loneliness slightly increased in the Care As Usual group whereas slight decreases were observed for the Experimental and Control Robot groups. These results can be interpreted in several ways. First, they indicate that using a culturally competent robot may be reasonably unlikely to increase loneliness compared to care as usual. Second, they indicate that, even over a short period of time (2 weeks), a slight reduction in loneliness may be observed. If these trends were to continue over time, these differences may become more meaningful. Third, if the full CARESSES culturally competent software was made available to the Experimental robot from the outset (rather than only during the second week of testing), the improvements in outcomes related to loneliness (and mental health) would likely have been even larger. However, it is clear that more work to improve the depth and impact of the system to drive down loneliness is required. Such improvements may tie-in with enhancements to the ‘social functioning’ part of the system, so that future systems can have a stronger focus on improving the social capital and circumstances of the individual [35].

Finally, with regard to perception of cultural competence, the results of the CCATool show that after one and two weeks of use, both the Experimental group’s robot and Control group’s robot were, overall, perceived to be reasonably culturally competent. Both versions of the robot were perceived to be more culturally competent after two weeks than compared to after one week, although the Experimental group showed greater improvement in scores overall. This may suggest that the changes made to the system after week 1, to increase cultural competence of the system (e.g. by enabling personalisation towards individual cultural values), had a positive impact upon perceptions of cultural competence and, as expected, a greater level of impact with the Experimental group participants. However, it may also be that increased scores over time were due to the participants’ growing understanding of how to use the system more as time passed. Or, of course, a combination of both these explanations.

There are a number of study limitations. Firstly, although the trial represents one of the most rigorous and largest of its kind to date, particularly with respect to cross-cultural assessments and intervention intensity, sample sizes were not statistically powered and may be viewed as fairly small (particularly the Japanese sample), consequently reducing generalisability. However, given that each test required two weeks, that only two robots were available to us, that on average only four residents per care home were eligible and agreed to participate and that, to achieve the study sample size spanned 10 months (across two countries) with many costs, collecting larger samples was not feasible within the overall project timescale. Furthermore, even though every attempt to control key potential confounding variables was made, and that gender-based stratified random group allocation was employed, it is likely that some confounding variables were present. Further, a likely source of bias was that participants using a robot were made aware that they were being audio and video monitored by researchers which may have inhibited or influenced some of their conversations and interactions with the robot. This was a bias that the research team could not fully overcome given that it was critical to monitor participants to ensure their safety and to intervene when technical problems occurred. Another potential bias could have been that some participants in Control Group 2 who wanted a robot but did not receive one had their mood negatively affected by being aware of their peers receiving one. Finally, it should be noted that this trial was based on a particular type of population, namely older adults residing in care settings without severe dementia, severe mental health problems or severe frailty (that would require hospitalisation). Therefore, inferences made on the potential impact of the system on other populations based upon the current system should be treated with caution, particularly older adults with severe levels of dementia and frailty.

Overall, the CARESSES trial represents one of the largest attempts to explore the impact of autonomous social robots on the health and wellbeing of older adults within social care settings, and the first to assess the role of culturally competent systems. Despite the study limitations, it can be cautiously concluded that the CARESSES system did not fare worse than care as usual in most of the study outcome measures and, particularly in relation to mental health and emotional wellbeing, it was able to produce significantly better outcomes. Further research is needed to confirm and build upon these findings, particularly those driven by statistically powered sample sizes, and to assess whether and how trends change beyond a 2-week period only.
